# iGWAS: Image-based genome-wide association of self-supervised deep phenotyping of retina fundus images

**DOI:** 10.1371/journal.pgen.1011273

**Published:** 2024-05-10

**Authors:** Ziqian Xie, Tao Zhang, Sangbae Kim, Jiaxiong Lu, Wanheng Zhang, Cheng-Hui Lin, Man-Ru Wu, Alexander Davis, Roomasa Channa, Luca Giancardo, Han Chen, Sui Wang, Rui Chen, Degui Zhi

**Affiliations:** 1 Department of Molecular and Human Genetics, Baylor College of Medicine, Houston, Texas, United States of America; 2 School of Biomedical Informatics, The University of Texas Health Science Center at Houston, Houston, Texas, United States of America; 3 School of Public Health, The University of Texas Health Science Center at Houston, Houston, Texas, United States of America; 4 Department of Ophthalmology, Stanford University School of Medicine, Stanford, California, United States of America; 5 Department of Ophthalmology and Visual Sciences, University of Wisconsin, Madison, Wisconsin, United States of America; 6 Human Genetics Center, Department of Epidemiology, Human Genetics and Environmental Sciences, School of Public Health, The University of Texas Health Science Center at Houston, Houston, Texas, United States of America; Washington State University, UNITED STATES

## Abstract

Existing imaging genetics studies have been mostly limited in scope by using imaging-derived phenotypes defined by human experts. Here, leveraging new breakthroughs in self-supervised deep representation learning, we propose a new approach, image-based genome-wide association study (iGWAS), for identifying genetic factors associated with phenotypes discovered from medical images using contrastive learning. Using retinal fundus photos, our model extracts a 128-dimensional vector representing features of the retina as phenotypes. After training the model on 40,000 images from the EyePACS dataset, we generated phenotypes from 130,329 images of 65,629 British White participants in the UK Biobank. We conducted GWAS on these phenotypes and identified 14 loci with genome-wide significance (p<5×10^−8^ and intersection of hits from left and right eyes). We also did GWAS on the retina color, the average color of the center region of the retinal fundus photos. The GWAS of retina colors identified 34 loci, 7 are overlapping with GWAS of raw image phenotype. Our results establish the feasibility of this new framework of genomic study based on self-supervised phenotyping of medical images.

## Author summary

Imaging genetics is a research field focused on understanding how genetic variations influence observable traits or diseases that can be visualized through medical imaging. Previous studies in imaging genetics have mostly relied on traits identified by human experts. In this study, we used a self-supervised contrastive learning algorithm that automatically identifies features in fundus images that are unique to each individual yet consistent between their left and right eyes. These features are represented as a set of 128 numbers that can quantitatively describe the characteristics of each individual’s fundus images without human labeling bias. We then conducted genome wide association studies (GWAS) to explore the associations between these features and genetic variations using paired fundus images and genetic data from the UK Biobank. Our findings indicate that these features are linked to genetic signals associated with eye measurements, eye diseases, pigmentation, and vessel development. Additionally, we found that GWAS of this set of features identified new genetic signals not detected in GWAS only using retina color. These results establish the feasibility of this self-supervised phenotyping approach for imaging genetics studies.

## Introduction

Although genome-wide association studies (GWAS) have successfully identified thousands of genetic associations, most existing GWAS are based on a set of predefined phenotypes. While these phenotypes encode valuable biomedical knowledge, they are also biased by current clinical practice and epidemiological studies. In addition, as the granularity of phenotype code is often limited, it is often not sufficient to capture the complexity of human physiology and pathology in their entirety. Therefore, deriving new phenotypes beyond expert curation would enable the discovery of new genetic associations.

Medical imaging is a rich resource for phenotype discovery. Through rapid technological advancements, modern medical imaging offers unprecedented details about a patient’s physiological condition and can be a high-content phenotyping modality. Most existing imaging GWASs have leveraged imaging-derived phenotypes (IDPs) [1–3]. These IDPs were typically designed by imaging experts and generated by special-purpose image processing pipelines. Recently, machine learning, especially supervised deep learning (DL), is used to automatically generate IDPs [4–6]. These methods were trained by learning from data labeled by experts and identified new loci in GWAS [1,2,7]. However, although supervised DL can vastly improve the efficiency of image labeling, it fails to provide phenotypes beyond those defined by experts. In addition, although these phenotypes are derived for medical practice, clinical decision processes, and natural-language-based reporting, they often do not comprehensively capture the imaging content. There are limitations to the amount of information a human eye can extract from images. Many meaningful imaging features, some of which might be used implicitly by physicians, may not be verbalized in medical reports. In addition, there may be physiologically informative features that are present in the image but are completely missed or ignored by readers. For example, Google’s DL algorithm extracted novel features from retinal images, such as age, gender, and smoking status, that are not readily apparent to expert human graders [8]. Following studies identified features such as refractive error and anemia from retinal images [9,10]. These results suggest that additional information beyond human curation may be encoded within imaging data, and new methods are needed to extract such information.

Here, we have designed a new framework of genome-wide genotype-phenotype association study by performing self-supervised image-based genome-wide association studies (iGWAS). For phenotype discovery, instead of supervised learning that relies on labels from expert annotations, self-supervised deep learning is applied to an image to capture its intrinsic contents [11–14]. Endophenotypes generated by the deep learning model are then subjected to GWAS to identify associated genomic loci.

We tested this new approach using human fundus images by deriving endophenotypes from the raw color fundus images, which likely capture the overall content of the image. We constructed a contrastive loss function over an Inception V3 architecture to learn a representation that captures the intrinsic retinal features of individuals. Our neural network outputs 128 endophenotypes representing the input image. After training on 40,000 images from EyePACS, our model generated phenotypes from 130,329 images of 65,629 British White participants in the UK Biobank. We then conducted GWAS analyses on the fundus image endophenotypes.

## Results

### Overall iGWAS framework

**Fig 1:**
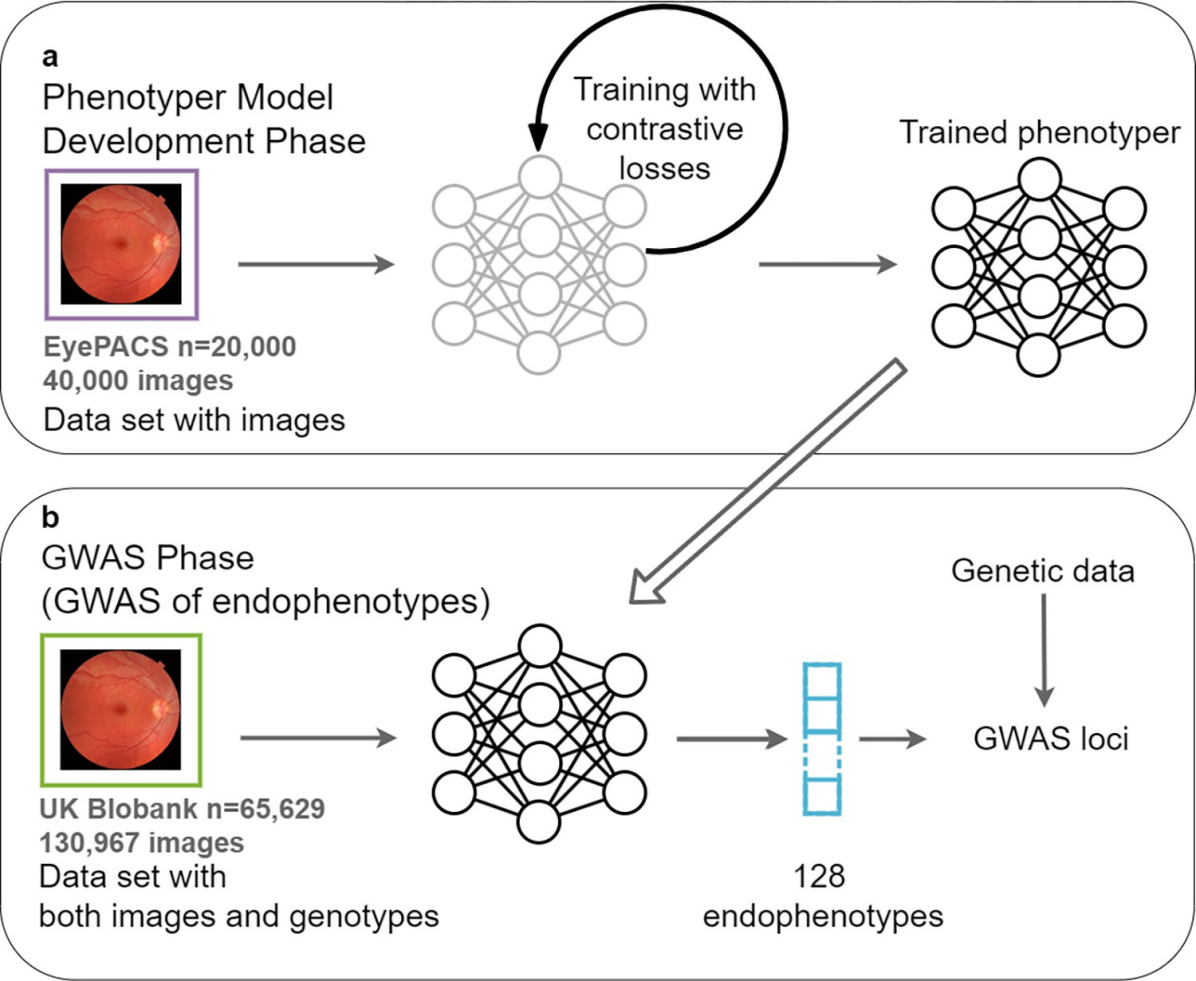
iGWAS of endophenotypes from retinal fundus images. (**a**) Using raw fundus images in EyePACS, we developed phenotyper neural networks that optimize contrastive losses; (**b**) Using the trained phenotypers, we generated 128 endophenotypes for each fundus image in the UK Biobank vision cohort and do GWAS on these endophenotypes to identified independent loci. The fundus photo in this figure is from Häggström M. “Medical gallery of Mikael Häggström 2014”. WikiJournal of Medicine. 2014;1(2). DOI:10.15347/wjm/2014.008. Licensed under Public Domain.

The core component of iGWAS is a phenotyping (encoder) neural network that generates endophenotypes, which are in turn associated with genotypes by GWAS (an example of iGWAS for retinal images is shown in **Fig 1**). Distinct from traditional phenotypes labeled by experts or by AI trained via supervised learning, iGWAS’s encoder network is trained by self-supervised learning to discover new phenotypes. We thus named it as **S**elf-**Su**pervised **P**henotyp**er** (SSuPer). Popular self-supervised learning losses, such as contrastive losses [11,13,15] and reconstruction losses [16], are used to extract coherent and biologically relevant features of individuals. We used a contrastive loss to learn features that are consistent between the images from the same person. The resulting “embedding vector,” the output of the encoder, is treated as “endophenotypes” for downstream GWAS analysis.

The iGWAS approach is executed in two phases: the model development phase and the GWAS phase. In the model development phase, a “phenotype development set” is used to train the embedding network. The phenotype development set is a collection of images from individuals, whose genotype data are not needed. The result of the model development phase is a trained neural network model, SSuPer, that can transform an input image into a set of self-supervised image-derived phenotypes (SS-IDPs). In the GWAS phase, the trained SSuPer from the model development phase is used to generate SS-IDPs for images from the “GWAS set,” a dataset containing both images and genotypes of a different cohort of individuals. The SS-IDPs are then tested for association with genome-wide markers.

### Overall data analysis strategy for generating endophenotypes from fundus images

In this study, we designed and implemented the iGWAS approach to encode retinal features from fundus images. For the phenotype development set, we used data from EyePACS, a large public collection of 88,702 fundus images (see **Methods: dataset extraction**). After quality control (see **Methods: Image quality control**), 40,000 top quality images were used (**S1 Table**). For the GWAS set, we used fundus images and genotype data of 65,629 British White UK Biobank participants. Although the demographics of the EyePACS and UK Biobank cohorts do not match exactly, we reasoned that some characteristics of their fundus images should be similar, so we expect the features learned from EyePACS can be generalized to UK Biobank.

First, the EyePACS fundus images are directly fed into the encoder neural network to generate raw image endophenotypes. A convolutional neural network (CNN) based on the Inception [17] architecture is used because it is proven to deliver good results for modeling images.

We found some of the endophenotypes strongly correlated with the color in image derived endophenotypes. Therefore, to account for the “retinal color,” defined as the average intensities of the red, blue, and green channels of the central patch of the fundus image, were considered as additional phenotypes in subsequent analysis (see **Methods**). While the definition of retinal color may not fully account for change in illumination, texture of the retinal pigment epithelium, retinal lesions, and optic disk, it captures coarse-grained information of the retinal background. We conducted GWAS analyses for the three sets of phenotypes: 128 raw image endophenotypes and 3 retina colors (RGB channels). To aid in interpretation of the endophenotypes, we conducted univariate and correlation analyses among endophenotypes and between endophenotypes and relevant eye phenotypes. The overall pipeline is shown in **S1 Fig**.

### Design of encoder network that captures coherent features of fundus images from the same person

**Fig 2:**
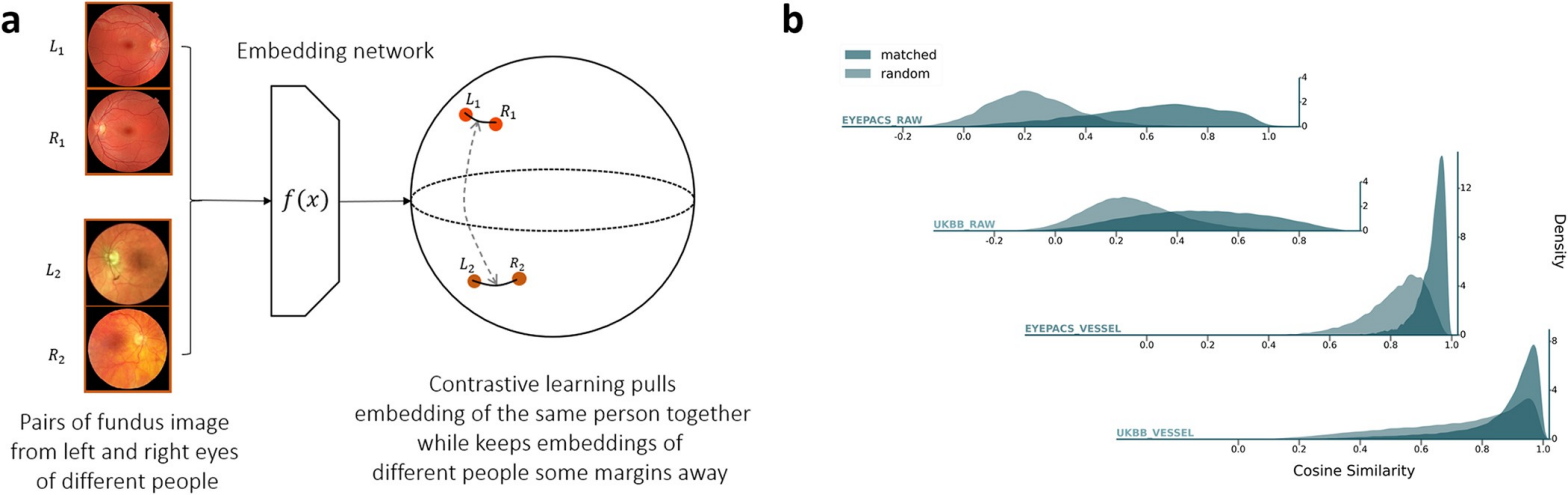
Contrastive loss for deriving phenotypes coherent across images from the same person. **(a)** Contrastive loss is designed to map images from the same person to be closer while keeping images from different persons apart. The top fundus photo pair is from Häggström M. “Medical gallery of Mikael Häggström 2014”. WikiJournal of Medicine. 2014;1(2). DOI:10.15347/wjm/2014.008. Licensed under Public Domain. The bottom fundus photo pair is adapted from Chalam KV, Chamchikh J, Gasparian S. “Optics and utility of low-cost smartphone-based portable digital fundus camera system for screening of retinal diseases”. Diagnostics. 2022 Jun 20;12(6):1499, licensed under CC BY 4.0. Available at: https://www.mdpi.com/diagnostics/diagnostics-12-01499/article_deploy/html/images/diagnostics-12-01499-g005.png. (Accessed: 2024-04-03). (**b**) The trained endophenotype vectors for fundus image embedding of the same persons reflect the design of contrastive learning in both the training set (EyePACS) and the test sets (UKBB). The distributions of the matched pairs (images from the same person) and the random pairs are separated. The distributions were estimated using Scott’s kernel with an additional multiplicative factor of 0.5 to smooth the curve.

To generate an embedding vector that represents the inherent biological features of an individual, we leverage a self-supervised metric learning approach that was described in ArcFace [18], a widely adopted algorithm that is used to extract features for developing human face recognition methods, with some technical modifications detailed in methods (see **Methods: Embedding neural network**). Inception v3, which has been demonstrated to be capable of capturing complex information within fundus images, was used as a backbone architecture for the metric learning [19]. The output of our embedding network was designed to be a 128-dimensional vector, based on previous work showing that 128-dimensional vectors are sufficient to represent complex datasets [20,21]. Our ArcFace loss function is a contrastive loss that first projects the embedding vector to the unit sphere and then optimizes the contrast between the embeddings from the eyes of the same person and the embeddings from different people by minimizing the angular distance between the embeddings of left and right retinas from the same individual while keeping the embeddings from different individuals at least some margins apart (**Fig 2**). We reasoned that if the trained model manages to capture real biologically relevant features, embeddings between an individual’s left and right fundus images should be more similar than those from different individuals, previous work also showed that genetic relatedness can be estimated from pairs of fundus images [22]. Details of model design and training are described in the **Methods: Embedding neural network**.

### Training of encoder networks

For 88,724 images from EyePACS, 54,992 passed our quality filter network (quality score > 0.5) (see **Methods**). 40,000 top quality images (quality score > 0.95) were selected as we reasoned that this balance point of sample size and sample quality is sufficient for training the main SSuPer network. The characteristics of the EyePACS dataset are shown in **S1 Table**.

To verify the performance of the SSuPer embedding network, we compared the matched pairs (left and right eye of the same person) and random pairs (**Fig 2B**). As expected, there is a clear separation in the distribution of cosine distance between matched pairs and random pairs (see **S2 Table** for quantification). The matched pairs are more similar in EyePACS than that in UK Biobank’s, indicating some level of domain shift. Although less than that of EyePACS, the separation of matched and random pairs was clearly observed in UK Biobank, indicating that the embedding models are transferable and indeed capture the intrinsic features of the fundus images. Therefore, we decided to directly apply the embedding networks trained using EyePACS to the UK Biobank data without fine-tuning. Of note, we observed a weak cone effect that the cosine similarity between any pair of embeddings is centered around 0.2, which is a general phenomenon for deep neural networks [23].

### Descriptive analysis of endophenotypes in UK Biobank fundus photos

We conducted our analysis using 65,629 British White participants from the UK Biobank who had available fundus images (see **Methods: Dataset extraction**). For each participant, we chose the first image for each eye, resulting in 130,329 images. Basic demographic description of this dataset is shown in **S3 Table**. Retina colors were also extracted as phenotypes. The central patch of the image (the fovea region) was used because it has more pigment and of low vessel density, providing a cleaner estimate of the retinal color (see **Methods: Extracting retina color and color GWAS**).

**Fig 3:**
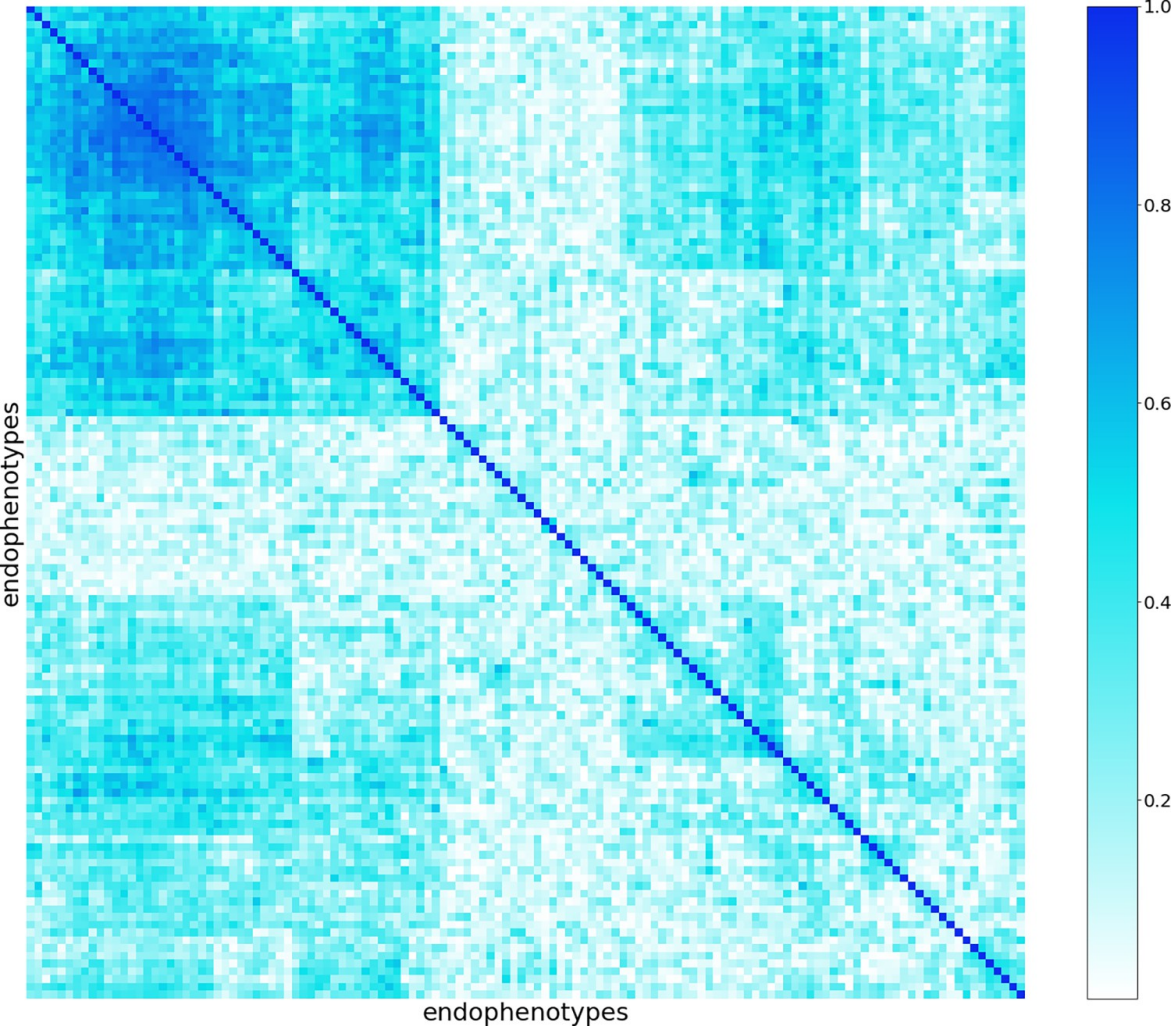
Absolute correlations among 128 image-derived endophenotypes. Some correlations are observed by hierarchical clustering.

Univariate distributions of the endophenotypes generated by our embedding networks is shown in **S2 Fig**. We found that most endophenotypes have unimodal bell-shaped distributions. Meanwhile, examining their pairwise correlations showed that endophenotypes have strong internal correlations (**Fig 3**).

### iGWAS: GWAS of endophenotypes

To identify genetic factors associated with endophenotypes, GWAS was performed for each of the 128 dimensions from all 130,329 images using linear mixed models as implemented by BOLT-LMM [24], adjusted by age, sex, and ancestral principal components (PCs) (see **Methods: Endophenotype GWAS**). Analyses were conducted separately for the left and right retinal images. Their results were not meta-analyzed because the endophenotypes of the two eyes may be correlated due to training. Instead, we pooled the results from the two eyes and took the intersection of the significant hits, and only the more significant p-value between the two eyes was reported. Since the endophenotypes were derived without the direct use of any genetic information, we expected there to be minimal genomic inflation for the GWAS. Indeed, we observed that the genomic inflation factor was well-controlled (λ_GC_≈1) (**S3 Fig**), though some endophenotypes had slightly higher (1.099) inflation factors, indicating potential polygenic genetic architecture.

**Fig 4:**
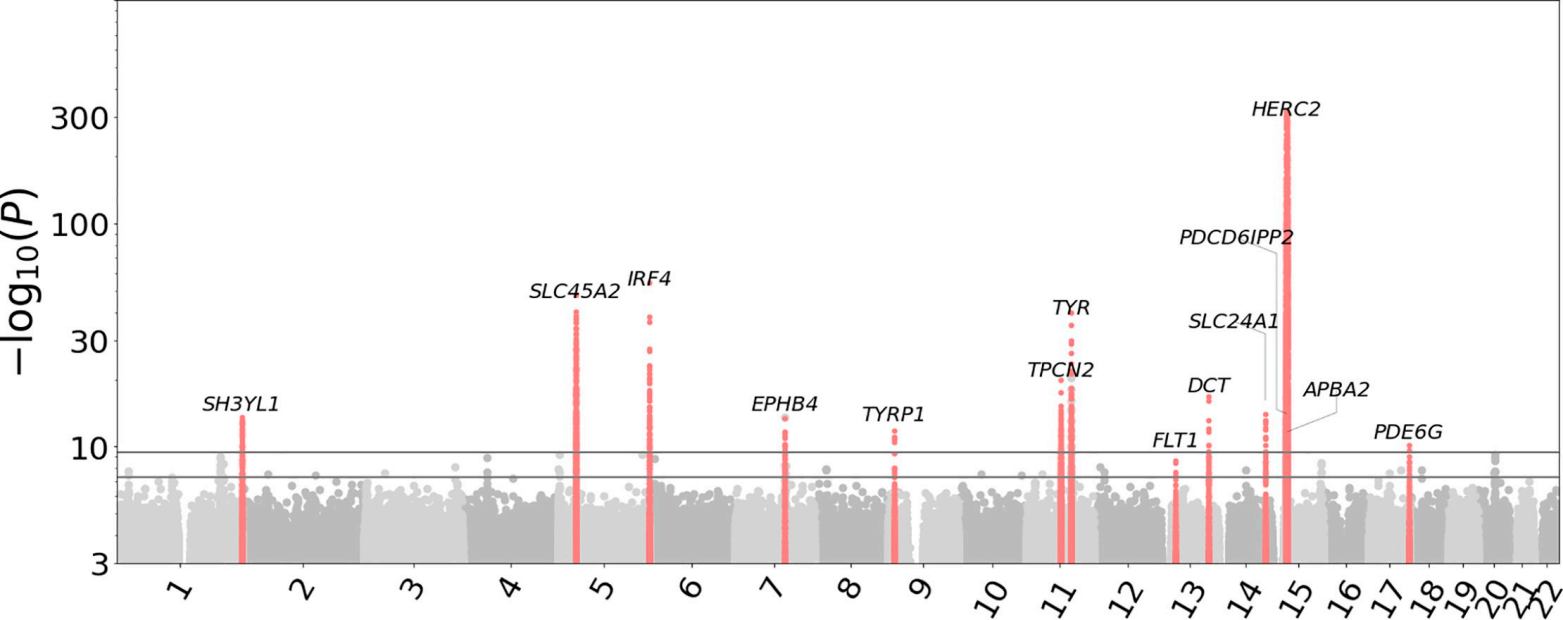
Aggregated Manhattan plots of 128 image endophenotypes. The two horizontal lines indicate significance levels set for individual GWAS (p = 5 × 10^−8^) and all phenotypes (p = 5 × 10^−8^/128). The red peaks are the image endophenotype associated loci that satisfy selection criteria defined in **Methods: Endophenotype GWAS**. Full SNP-endophenotype association table for these peaks is available at S5 Table.

We identified 2,150 SNP-endophenotype pairwise association signals from 113 SNPs (**S4 and S5 Tables**) showing genome-wide significance (p-value<5×10^−8^) (**Fig 4**). These SNPs were clustered into 14 independent loci (**Table 1**) (see **Methods: Endophenotype GWAS**).

**Table 1:** 14 loci significantly associated with raw image endophenotypes. BP is in GRCh37 coordinate. The raw_PVAL column contains the most significant p-value (min P) of the image endophenotype GWAS among all endophenotypes at each locus. Non-significant p-values are omitted. The color_PVAL contains the most significant p-value of the retina color GWAS among 3 color channels, and left empty if not significant. Candidate genes for each locus were annotated based on their distance from the leading SNPs and annotated function.

SNP	CHR	BP	REF/ALT	ALTFREQ	raw_PVAL	color_PVAL	GENE
rs17713396	2	227,201	C/T	0.349497	3.10E‐14	8.40E‐09	SH3YL1
rs16891982	5	33,951,693	G/C	0.022837	2.20E‐48	8.20E‐38	SLC45A2
rs12203592	6	396,321	C/T	0.204664	7.00E‐55		IRF4
rs117756744	7	100,277,212	G/A	0.021396	4.10E‐14		EPHB4
rs1408799	9	12,672,097	C/T	0.308287	1.40E‐12		TYRP1
rs72928978	11	68,831,364	G/A	0.109878	1.10E‐20	6.00E‐18	TPCN2
rs1847134	11	89,005,253	A/C	0.334505	2.10E‐40		TYR
rs12428170	13	29,171,890	G/A	0.158271	2.40E‐09	4.20E‐25	FLT1
rs9561576	13	95,157,722	C/T	0.313216	1.60E‐17		DCT
rs12896399	14	92,773,663	G/T	0.453193	8.60E‐15	1.20E‐23	SLC24A1
rs1129038	15	28,356,859	T/C	0.215305	0	0	HERC2
rs116388828	15	29,045,316	C/A	0.433252	8.80E‐13		PDCD6IPP2
rs12912104	15	29,332,198	G/A	0.251294	2.30E‐10		APBA2
rs8908	17	79,617,871	A/G	0.378218	2.60E‐10	7.20E‐18	PDE6G

The mean and standard deviation of the heritability from LD score regression (**S4 Fig and S6 Table**) of the image endophenotype is 0.04 and 0.05 (t-test p-value = 1.8×10^−37^).

We queried the GWAS Catalog for the 14 loci (Methods: Querying GWAS Catalog, S7 Table) and found most of them are associated with eye measures, eye diseases, pigmentation and conditions such as diabetes or cardiovascular diseases. The association with the diseases might be explained by the effects these conditions have on the retina, diabetes, for instance, is a known risk factor for diabetic retinopathy, and retinal vessel abnormalities can be indicative of hypertension and increased risk of cardiac events [25]. Three of the 14 loci showed no previous genetic association with these categories, yet they are assigned to the genes *DCT, PDE6G and EPHB4*, which are related to pigmentation, eye diseases or vessels. *DCT* plays a role in melanin production in the retina and hence eye color related [26]. *PDE6G* encodes the gamma subunit of cGMP-phosphodiesterase and is associated with diseases such as night blindness and retinitis pigmentosa [27]. *EPHB4* is essential in vessel development [28] and modulation of *Ephb4* activity in the mouse retina was found to alter retinal neovascularization [29,30]. Genes at other loci, such as *HERC2/OCA2*, *TYRP*1 and *APBA2* [31,32], are also related to pigmentation and eye color, while *FLT1*’s role as a negative regulator of *VEGF* highlights its importance in retinal vessel development [33]. Eye diseases such as glaucoma, diabetic retinopathy and age-related macular degeneration also appear to be associated with the loci in our GWAS Catalog queries (**S7 Table**), suggesting a potential link between the learned endophenotypes and the eye diseases.

### GWAS of retina color

Pigmentation of the human body, such as hair, skin, and iris, is strongly influenced by genetics. As the color of the human retina is influenced by factors such as the level of pigmentation of retinal pigment epithelium (RPE) and choroid blood vessels, we tested if genomic loci associated with retina color can be identified through genome association study of fundus images (**Methods: Extracting retina color and color GWAS**). While association of iris color has been conducted [34], no direct association studies of retinal color using fundus images have been conducted. In our study, significant genome-wide association (p<5×10^−8^, and intersection between hits from fundus images of left and right) was obtained for a total of 175 SNPs (**S8 Table** and **S5 Fig**) from 34 independent loci (**S9 Table**).

We found 13 out of the 34 retina color loci overlapped with previously reported GWAS loci for “hair color”[35], “eye color”[36], and “skin pigmentation”[36] in the GWAS Catalog (**S9 Table** and **S6 Fig**, see **S9 Table** for details), supporting the validity our approach. Interestingly, many genes from unique loci identified in this study can be linked to pigmentation pathways (**S9 Table**). For example, mutations in *FGFR3* lead to familial acanthosis nigricans, which results in skin pigmentation abnormalities [37]. In addition to pigmentation, it is interesting to note that 6 of the 34 loci overlap with loci previously reported to be associated with macular thickness (**S9 Table**), including *DCDC1*, *TPCN2*, *NCAM1*, *HERC2*, *PDE6G*, and *WNT7B*.

### Genetic correlation analyses of endophenotypes

To further interpret these endophenotypes, we correlated them with other traits that are related to retinal phenotypes. We conducted genetic correlation using summary statistics as they are easier to access and are suggested to be a good surrogate for phenotypic correlation [38,39]. We included traits that either have GWAS hits near the endophenotype GWAS loci (within 250 kb) or are known to be related to retinal or corneal disorders, and whose genetic summary statistics for UK Biobank data are available (see **Methods: Genetic correlation**). Corneal phenotypes (H15-H22 Disorders of sclera, cornea, iris and ciliary body and H18 Other disorders of cornea from GeneAtlas [40]) were included because they may affect refractive error, which can have a detectable effect on the fundus images. We found that many endophenotypes are genetically correlated with skin/hair pigmentation and retinal color after Bonferroni correction (corresponds to p-value threshold of 0.05/128). Other nominally significant genetically correlated pairs (not significant after Bonferroni correction) include correlations between endophenotypes and cardiovascular disease, diabetes, lung function and blood pressure (**S10 Table**).

We also correlated (phenotypically and genetically) the endophenotypes with fundus background color and found that they are strongly correlated. (**S10 Table**).

## Discussion

Our work is one of the first proof-of-concept studies of a self-supervised learning-based phenotype discovery method for imaging GWAS. With no expert supervision, our method was able to extract endophenotypes and identify genes relevant to the retina, including retina colors, retinal vessel development, and eye diseases such as glaucoma, diabetic retinopathy and age-related macular degeneration.

While there have been previous imaging GWAS on DL-based phenotyping, they either used expert-defined phenotypes [1,41] or clustering of dense representational vectors into subtypes. We directly use the dense vectors, which contain more information than the subtype cluster labels, as phenotypes. There are a handful of studies that use final or intermediate layers of the neural network as phenotypes, but these networks were trained in a supervised fashion using external labels (e.g., age [42] or eye diseases [43]) or via transfer learning [44]. Of note, there is another contrastive learning approach, ContIG, for phenotyping the retina fundus images by maximizing cross-modality matching between the image part and the genetic part of the same individuals [45]. iGWAS does not require data sets with both images and genetic data to train the encoder, and may have a wider range of applicability.

Other architectures or unsupervised learning algorithms are also possible options. Auto-encoder and its variants are generally a good choice for representation learning. However, the auto-encoder would try to capture all variations of the data but is practically challenging to align fundus images to remove irrelevant variations such as rotation or translation because the vessels can assume flexible shapes (Some models aim to learn disentangled latent representation, but studies [46] have shown that their performances are not reliable). We chose contrastive learning over auto-encoder because we thought it was important to learn a representation that is insensitive to some perturbations (random rotations and two eye differences) but focus more on patterns that are common to both eyes and hopefully be more heritable. In fact, in another work [47] of ours, we used auto-encoder to learn representations of the brain MRI imaging data because brain MRI can be well registered to remove scaling, rotation and translation.

In our study, each subject serves as a distinct class. Therefore, we have opted for Arcface-like methods which facilitate contrastive learning across batches by maintaining a template representation for each class. These methods also promote inter-class separation by introducing a margin on top of the cross-entropy loss. This approach is advantageous for our purposes over current state-of-the-art methods such as BYOL or SimCLR, which typically necessitate large batch sizes that might not align with our computational budget. The Arcface-like loss has shown superior performance in the recent Kaggle challenges for representation learning [48,49].

Our iGWAS framework is flexible and can be adapted and extended in various ways in the future. To study retinal vasculature embedding, we can first have a segmentation step that generates vessel masks, and then subsequently derive the vasculature embeddings from these predicted vessel mask images. To capture other information in fundus images, such as the morphology of the optic disc, hemorrhages, exudates, or the pigmentation level, alternative preprocessing/segmentation steps may be applied, or this process can be completely skipped. Also, while the pair of eyes of an individual are natural “biological replicates” for our ArcFace-like approach, our approach may be extended to images without replicates, via current approaches for contrastive learning [13]. Furthermore, to inject labels to make more specific phenotypes, one can use a hybrid approach that minimizes both supervised and self-supervised losses.

While we prioritize the proof-of-concept, there is room for further methodological improvements. For example, it is not completely optimized to use the 128-dimensional vector as phenotypes. Moreover, the phenotyping model were trained in different datasets then directly deployed to the UK Biobank data so there may exist some distribution shift that we didn’t account for. We chose not to do domain adaptation on the UK Biobank data set to avoid false association signals due to information leaking. Addressing the distribution shift may improve the separation of endophenotype distances between matched and random pairs in the UK Biobank. In addition, lack of clear image interpretation of our endophenotype derived from self-supervised learning might be a major limitation. To probe the semantics of the learned embeddings, a common approach is to use the saliency methods such as smooth-grad [50] to find the important part of the input that affect the embedding or visualize a coarse-grained activation map using approaches such as class activation map [51]. Another approach would be pairing the encoding model with a decoding model that can reconstruct the image from the learned embeddings, with such a decoding model, we can perturb the embeddings and look at the changes in the reconstructed images to gain insights into the model’s features. Recognizing the lack of interpretability, future work is needed to engage image interpretation methods to identify relevant image features. Moreover, our retina color GWAS uses RGB color, which may be susceptible to change in illumination. Defining retina color in other color spaces may further improve the detection power. Furthermore, we observed that certain loci found by retina color are not detected by the deep learning-based method. It might be that the average intensity around the fovea region may not be the most effective feature for distinguishing between subjects and recognizing the left and right eyes of the same person, and neural networks with contrastive learning tend to neglect unessential features during the training [52]. Further research is needed to find different tasks that could enable the capture of more genetic signals.

Our self-supervised learning method can be applied to other medical imaging domains to aid gene discovery. While retina images of two eyes of a person are natural “monozygotic twins”, up to a flip, that share a same genetic profile, this approach is applicable to other image modalities that have similar symmetry, e.g., kidney, skeletal, or even brain hemispheres. Moreover, this approach is applicable to images with repeated measures.

We explored retinal colors in this work. Retinal color may affect the recent GWAS [7,41,53,54] on AI-based automatic extracted phenotypes from fundus images including optic nerve head morphology, retinal vessel measurements which also identified the *HERC2*/*OCA2* locus as the strongest hit. In addition, as in any association study, genetic loci identified in our study could be due to secondary effects of other hidden confounding factors. For example, eye conditions such as refractive error could affect the appearance of the fundus image. In addition, other factors such as retinal background texture were not considered. More sophisticated representation learning with disentanglement may be used to control for these correlations [55,56]. Therefore, to establish causality relationship between the gene loci with the phenotype, further investigation, such as follow up functional experiments presented in our study, is essential.

While this work is not focusing on retinal vessels, some genes relevant to vessel development showed up in our GWAS hit list. This is because retinal vessels are prominent features of the fundus images. Retinal vessels are very relevant to eye diseases such as diabetic retinopathy and age-related macular degeneration and are often the focus of imaging genetics studies. However, if the goal is to study retinal vessels, some image processing that enriches the signal to noise ratio of vessels might be needed. This will be an interesting direction for future research.

In sum, the benefit of self-supervised-learning-derived phenotypes is that no external training labels are required. This frees up the burden of complicated and expensive labeling and makes our approach applicable to any large collection of images. As we leverage big datasets to improve our understanding of diseases, self-supervised methods are needed to efficiently extract meaningful information from medical images. We predict that iGWAS as a general phenotype discovery approach will be a fruitful research avenue.

## Methods

### Ethics statement

Our analysis was approved by University of Texas Health Science Center at Houston committee for the protection of human subjects under No. HSC-SBMI-22-0744. UK Biobank has secured written informed consent from the participants in the use of their data for approved research projects. UK Biobank data was accessed via approved project 24247.

### Data set extraction

The DRIMDB dataset [57], was downloaded on 2018/11/26 from https://www.researchgate.net/publication/282641760_DRIMDB_Diabetic_Retinopathy_Images_Database_Database_for_Quality_Testing_of_Retinal_Images. We used it as part of the training set to train the quality control network as it contains images with quality labels. It contains 69 bad quality fundus images and 125 good quality fundus images.

The EyePACS dataset (accessed in 2018/11/02) was downloaded from Kaggle.com. It contains fundus images from both healthy subjects and subjects with different grades of diabetic retinopathy. 35,126 Kaggle training set images and 53,576 Kaggle test set images were combined. The demographic characteristics including age, sex, and ethnicity of individual images were undisclosed.

The UK Biobank data was accessed via approved project 24247. We conducted our analysis on over 65,629 British White (self-reported white British (field: 21000) and genetically identified as Caucasian (field: 22006)) participants from the UK Biobank who had fundus images available (field: 21015 and 21016). For each participant, we chose the first image for each eye, resulting in 130,329 images. Genetic data as genotyped by Applied Biosystems UK BiLEVE Axiom Array (field: 22438) and imputed (field: 22828) [58] were downloaded. The fundus images in the UK Biobank data were taken using the TOPCON 3D OCT 1000 Mk2 alongside with the optical coherence tomography (OCT) imaging data. The data were collected in two phases: the initial assessment visit (2006–2010) at which participants were recruited and consent given and the first repeat assessment visit (2012–13). The size of each fundus image is 1536x2048 pixels.

### Image quality control

We trained a neural network to automatically assess the quality of the fundus images. Since the DRIMDB does not contain enough labelled images, we manually labeled 1,000 fundus images of good and bad quality from the EyePACS dataset and combined them with the DRIMDB dataset as the training set. An Inception v3 network [17] pretrained on ImageNet was downloaded and fine-tuned to classify qualities of different samples with early stopping. The quality assessment network outputs a score between 0 (bad) and 1 (good) to indicate the quality of the image, and it was trained using cross entropy loss. An image was defined as good quality if the output quality score of the network from that image was greater than 0.5.

The performance of the quality assessment network was validated on a subset of UK Biobank fundus images taken from white British subjects with diabetes mellitus (n = 7,683). A previously validated procedure was used to determine DM status based on self-reported DM diagnosis, use of DM medications and presence of DM complications [59]. We also used HbA1c > 6.5% as a criterion for identifying DM. Two ophthalmologists were asked to grade the image for the stage of diabetic retinopathy and determine if an image is of bad quality. A fundus image in this subset was classified as bad quality if both graders agreed that the quality of the image is poor. Comparing with this ground truth, the quality assessment network reached an AUC ROC of 92.14%. At 0.5 threshold, the positive predictive value was 0.9832, the negative predictive value was 0.4916, the sensitivity was 0.7155, and the specificity was 0.9574.

### Embedding neural network

The raw fundus images were fed to a network that uses the Inception v3 [17] backbone to produce a 128-dimensional embedding vector. The final fully connected layer of the Inception v3 network was replaced to produce a 128-dimensional vector. We adopted an approach similar to ArcFace [18]: Each subject is assigned a template embedding and the network is trained to minimize the angle between embeddings of different photos of a subject and his/her template while maintaining a margin between embeddings of a specific photo and templates of different subjects. Specifically, our loss function is: $L=\frac{1}{N}\sum_{i=1}^{N}\log\frac{e^{s\;\cos(\theta_{i}+m)}}{e^{s\;\cos(\theta_{i}+m)}+\sum_{i=1,i\neq j}^{N}e^{s\;\cos\theta_{j}}}$

where *N* is the number of samples, *θ*_(j)_ is the angle between the output of the network and the template of the *j*th sample, *m* is the margin, and *s* is the inverse temperature scaling factor. In our study, *m* is set to be 30 and *s* is set to be 0.5, which are the best performing hyperparameters on multiple face recognition datasets.

The embedding network was trained using 40,000 images from the EyePACS database (https://www.eyepacs.com/). The quality control network was used to score each image, and the top-ranked 40,000 images were taken. The right eye images were flipped for preprocessing, and random rotations were applied to add robustness. The training-testing split was 80/20. We also trained the embedding network with an additional task of classifying the grade of diabetic retinopathy. The weight ratio of these two tasks was 10 to 1. The network was trained using Adam optimizer [60] with a learning rate of 1×10^−4^ for 500 epochs on RTX 2080Ti and A100, and the model with the lowest test set loss was selected. Source code is available at https://github.com/ZhiGroup/iGWAS.

### Endophenotype GWAS

The genome-wide scans for UK Biobank were conducted over 658,720 SNPs that were directly genotyped by UK Biobank Axiom Array (field: 22438). To control for confounding factors due to ethnicity, we only included individuals of British white ethnicity (self-reported white British (field: 21000) and genetically identified as Caucasian (field: 22006)). The sample size was 65,629. We used all 130,329 images from this cohort without applying image quality control. The GWAS was performed with BOLT- LMM (Version 2.3.4) [24] on all 128 dimensions of the embedding vector using the linear mixed model association method (BOLT_LMM_INF) with age, sex, and the first 10 ancestral principal components as covariates. In total, we conducted 256 GWAS, one for each of the 128 endophenotypes from one eye. As a result, each variant had 256 p-values, 128 for the left and 128 for the right fundus images. A variant was selected if the minimum of the left 128 p-values and the minimum of the right 128 p-values both passed a threshold of 5×10^−8^. For each individual endophenotype for the left eyes, we used the typical 5x10^-8^ p-value threshold for GWAS, which incorporates a Bonferroni correction to adjust for the order of 1x10^6^ SNPs. We use the right eyes GWAS as replication and we also used the same 5x10^-8^ cutoff. In practice, we achieve this by running GWAS on either eyes and require that the association p-value for both eyes pass 5x10^-8^. Effectively, we were looking at the larger p-value of both eyes and comparing it to 5x10^-8^. This is a more conservative approach than a single phenotype GWAS. These selected variants from both eyes and all endophenotypes were then merged into independent loci if they are in linkage disequilibrium (r^2^>0.2) or within 250 kb from each other, a typical practice of the field [61,62].

### Extracting retina color and color GWAS

The traits of retina color were created as follows. The size of each UK Biobank fundus image is 1536x2048 pixels. Right eye fundus images were first flipped before cropping. The center patch of size 400x400 pixels around the fovea region, [600:1000, 800:1200], was cropped, and the average intensities of each of 3 channels (red, green, and blue) in this patch were taken as the quantitative traits. Since the fundus images of UK Biobank are mostly aligned as they are taken with unified protocol, the patches at the same location were comparable. In addition, the GWAS analyses were done on the same cohorts and using the same pipeline as in the endophenotype GWAS.

### Heritability and Genetic correlation

The heritability and genetic correlations were estimated using LDSC software (v1.0.1, 63). 1000 Genome European reference panel was used to calculate the heritability. We then selected several traits (**S10 Table**) to probe the endophenotypes and counted the number of GWAS loci that overlapped with traits from the GWAS Catalog (**S6 Fig**). The selection criteria were: (1) The previous GWAS hits of the trait fall within any iGWAS loci more than twice or the traits are related to retinal or corneal disorders, and (2) The summary statistics of the trait are available from either https://alkesgroup.broadinstitute.org/UKBB/UKBB_409K/ or  http://geneatlas.roslin.ed.ac.uk. To our knowledge, these are the only publicly available summary statistics computed by linear mixed models.

### Querying GWAS Catalog

For each independent locus, the range from the first to the last significant SNP was first transformed using LiftOver, then a range query was performed with the range plus 250 kb flanking regions on the GWAS Catalog database to identify previous associations (**S7 Table**).

## Supporting information

S1 Fig The overall pipeline of the study. (TIF)

S2 Fig Univariate distribution of 128 endophenotypes derived from fundus images. (TIF)

S3 Fig Genomic inflation factors of each dimension of SSuPER endophenotypes from fundus images. (TIF)

S4 Fig Scatter plot for the heritabilities of image endophenotypes directly estimated by the LD score regression. (TIF)

S5 Fig Fundus background color Manhattan plot. (TIF)

S6 Fig Venn diagram of the number of overlapping loci between fundus background color and other traits. (TIF)

S1 Table Descriptive Summary of diabetic retinopathy levels of the first 40000 fundus images of the EyePACS dataset, ranked by the image quality. (XLSX)

S2 Table Jensen-Shannon Distances and Jaccard Indices (intersection over union) between cosine similarity distribution of random and paired samples. (XLSX)

S3 Table Descriptive Summary of demographic factors of the UK Biobank cohort, including white British with retina fundus images. (XLSX)

S4 Table List of 113 SNPs associated with any of the 128 endophenotypes derived from the raw images of both eyes. (XLSX)

S5 Table SNP-endophenotype pairs showing significant associations with both left and right eyes. (XLSX)

S6 Table Heritability of the raw image endophenotype. (XLSX)

S7 Table GWAS Catalog query result for each locus. (XLSX)

S8 Table List of 175 SNPs associated with retina color of both eyes. (XLSX)

S9 Table List of 34 loci associated with retina colors of both eyes. (XLSX)

S10 Table Genetic correlation between raw image endophenotypes and other traits. (XLSX)
