# Supplementary figures and images for: iGWAS: Image-based genome-wide association of self-supervised deep phenotyping of retina fundus images
Source: PLoS Genet. 2024 May 10;20(5):e1011273. doi: 10.1371/journal.pgen.1011273 (PMC11111076; doi:10.1371/journal.pgen.1011273)

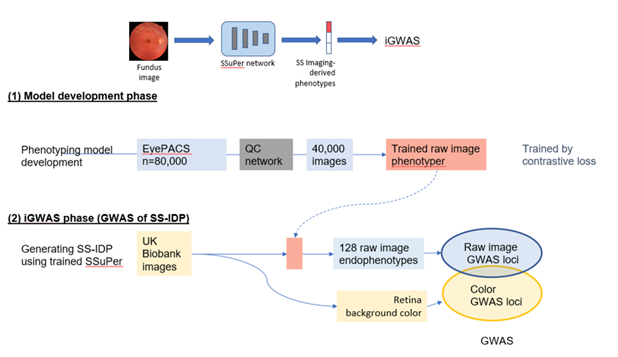

Supplement: S1 Fig — (TIF) [file pgen.1011273.s001.tif]

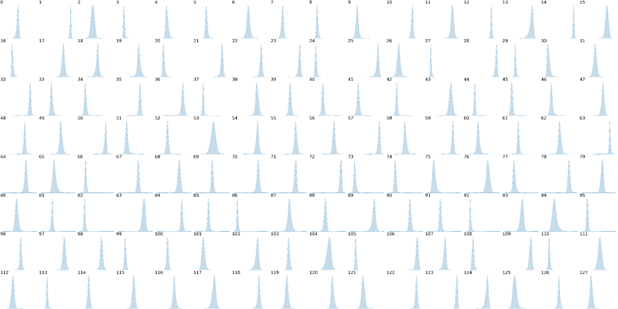

Supplement: S2 Fig — (TIF) [file pgen.1011273.s002.tif]

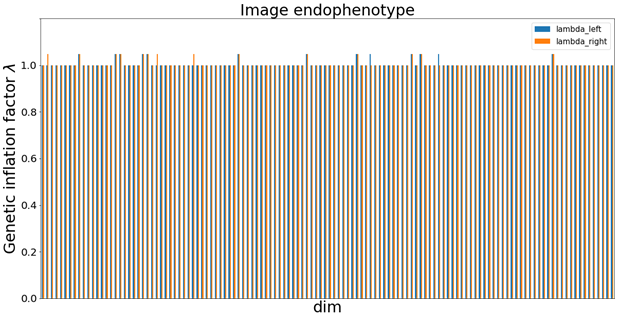

Supplement: S3 Fig — (TIF) [file pgen.1011273.s003.tif]

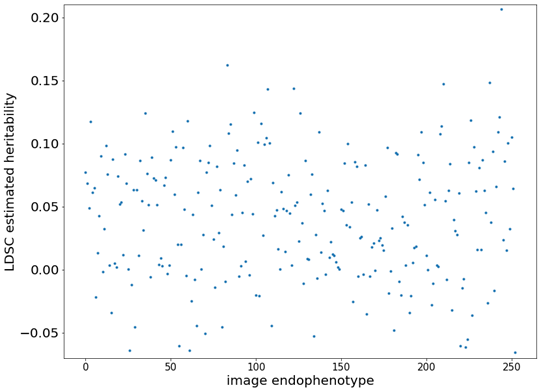

Supplement: S4 Fig — (TIF) [file pgen.1011273.s004.tif]

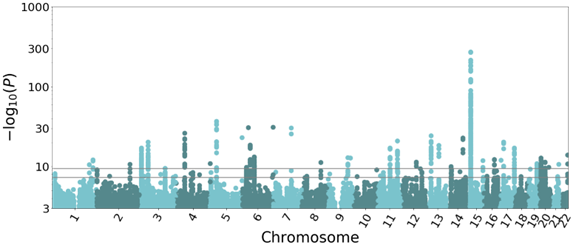

Supplement: S5 Fig — (TIF) [file pgen.1011273.s005.tif]

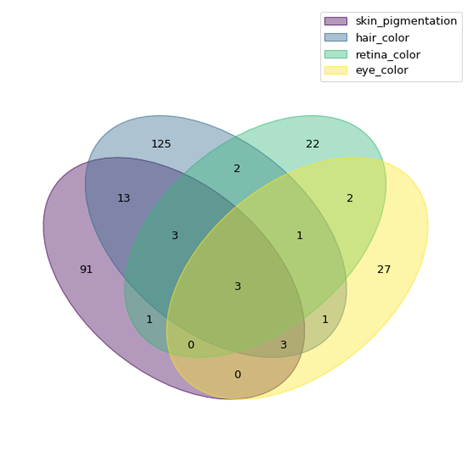

Supplement: S6 Fig — (TIF) [file pgen.1011273.s006.tif]
